# A novel immunogenomic signature to predict prognosis and reveal immune infiltration characteristics in pancreatic ductal adenocarcinoma

**DOI:** 10.1093/pcmedi/pbac010

**Published:** 2022-04-25

**Authors:** Ang Li, Bicheng Ye, Fangnan Lin, Yilin Wang, Xiaye Miao, Yanfang Jiang

**Affiliations:** Key Laboratory of Organ Regeneration & Transplantation of the Ministry of Education, Genetic Diagnosis Center, The First Hospital of Jilin University, Changchun 130021, China; School of Clinical Medicine, Medical College of Yangzhou Polytechnic College, Yangzhou 225100, China; Key Laboratory of Organ Regeneration & Transplantation of the Ministry of Education, Genetic Diagnosis Center, The First Hospital of Jilin University, Changchun 130021, China; Key Laboratory of Organ Regeneration & Transplantation of the Ministry of Education, Genetic Diagnosis Center, The First Hospital of Jilin University, Changchun 130021, China; School of Clinical Medicine, Yangzhou University, Yangzhou 225100, China; Key Laboratory of Organ Regeneration & Transplantation of the Ministry of Education, Genetic Diagnosis Center, The First Hospital of Jilin University, Changchun 130021, China

**Keywords:** pancreatic ductal adenocarcinoma, immune-related gene, clinical outcome, immunotherapy

## Abstract

**Background:**

The immune response in the tumor microenvironment (TME) plays a crucial role in cancer progression and recurrence. We aimed to develop an immune-related gene (IRG) signature to improve prognostic predictive power and reveal the immune infiltration characteristics of pancreatic ductal adenocarcinoma (PDAC).

**Methods:**

The Cancer Genome Atlas (TCGA) PDAC was used to construct a prognostic model as a training cohort. The International Cancer Genome Consortium (ICGC) and the Gene Expression Omnibus (GEO) databases were set as validation datasets. Prognostic genes were screened by using univariate Cox regression. Then, a novel optimal prognostic model was developed by using least absolute shrinkage and selection operator (LASSO) Cox regression. Cell type identification by estimating the relative subsets of RNA transcripts (CIBERSORT) and estimation of stromal and immune cells in malignant tumors using expression data (ESTIMATE) algorithms were used to characterize tumor immune infiltrating patterns. The tumor immune dysfunction and exclusion (TIDE) algorithm was used to predict immunotherapy responsiveness.

**Results:**

A prognostic signature based on five IRGs (*MET, ERAP2, IL20RB, EREG*, and *SHC2*) was constructed in TCGA-PDAC and comprehensively validated in ICGC and GEO cohorts. Multivariate Cox regression analysis demonstrated that this signature had an independent prognostic value. The area under the curve (AUC) values of the receiver operating characteristic (ROC) curve at 1, 3, and 5 years of survival were 0.724, 0.702, and 0.776, respectively. We further demonstrated that our signature has better prognostic performance than recently published ones and is superior to traditional clinical factors such as grade and tumor node metastasis classification (TNM) stage in predicting survival. Moreover, we found higher abundance of CD8+ T cells and lower M2-like macrophages in the low-risk group of TCGA-PDAC, and predicted a higher proportion of immunotherapeutic responders in the low-risk group.

**Conclusions:**

We constructed an optimal prognostic model which had independent prognostic value and was comprehensively validated in external PDAC databases. Additionally, this five-genes signature could predict immune infiltration characteristics. Moreover, the signature helped stratify PDAC patients who might be more responsive to immunotherapy.

## Introduction

Pancreatic ductal adenocarcinoma (PDAC) is one of the most lethal neoplasms with a median survival duration of <6 months and is usually diagnosed at a late stage.^1,2^ Epidemiology shows that PDAC has been the fourth leading cause of cancer-associated death in the world.^3^ Despite recent advances in various treatments for PDAC, including surgery, radiotherapy, chemotherapy, and targeted therapies, the prognosis for PDAC is still poor. Up to now, the prediction of PDAC prognosis has mainly depended on the histopathological diagnosis and tumor staging system. Traditional methods could not accurately predict the prognosis and help clinicians stratify patients with PDAC.^4^ Therefore, identifying the molecules that affect the prognosis and establishing a prognostic model is crucial for the management of PDAC patients.

Various immune-relevant gene (IRG) signatures are associated with the prognostic value and sensitivity of various therapeutic drugs.^5^ The expression of immune-related genes in the tumor region could also suggest the quality and abundance of immune infiltration in the tumor microenvironment (TME). PDAC is reported to be an immunogenic tumor. The purpose of immunotherapy can be achieved by targeting immune checkpoints.^6,7^ However, the clinical heterogeneity of patients is large and the prognosis is difficult to precisely assess. Thus, a detailed description of the IRGs and immune infiltration characteristics may benefit the development of novel precise biomarkers and better targeted immunotherapy. Several IRG signatures have been proved to improve the prognosis prediction for patients with PDAC.^8,9^ However, most studies had similar limitations, e.g. they were validated in only a few external independent cohorts (≤3 external cohorts). Additionally, few previous signatures explored the association with immunotherapy.

In this study, we constructed a novel five-genes signature that had independent prognostic value, and comprehensively validated it in two external PDAC databases [International Cancer Genome Consortium (ICGC) and Gene Expression Omnibus (GEO)], including four clinical cohorts. Receiver operating characteristic (ROC) curves further showed that the risk score has higher sensitivity and specificity and was superior to traditional clinical factors and other signatures recently published. Additionally, this five-genes signature could predict immune infiltration characteristics: abundance and dysfunction of CD8+ T and M2-like macrophages. All of these indicated that this signature can be used as a biomarker for prognostic prediction of PDAC and help stratify PDAC patients who might be more responsive to immunotherapy.

## Methods

Because our data were all downloaded from public databases there were no requirements for ethical approval. The study was conducted in accordance with the Declaration of Helsinki (as revised in 2013).

### Clinical samples and immune genes data collection

All transcriptome RNA expression matrices were obtained from The Cancer Genome Atlas (TCGA) database (https://portal.gdc.cancer.gov/) and Genotype Tissue Expression (GTEx) datasets (https://xenabrowser.net/), which contained 147 PDAC tumor samples and 167 normal pancreas samples respectively. For further analysis, the clinical data of PDAC patients were also downloaded. For the GTEx and TCGA datasets, the RNA-seq raw read count was converted to transcripts per kilobase million (TPM), then further normalized to log_2_ (TPM + 1). Additionally, in order to confirm the signature had a robust ability to distinguish prognosis, four external validation cohorts were included, including two independent ICGC datasets (ICGC-PACA-AU and ICGC-PACA-CA) and two microarray cohorts (GSE57495 and GSE62452) from GEO databases. Batch effects were removed by the surrogate variable analysis (SVA) algorithm.

In addition, IRGs were identified via the Immunology Database and Analysis Portal (ImmPort) (https://www.immport.org/). ImmPort is an open database with human immunology data, which can keep updating immunology data in an accurate and timely manner. This also facilitates accurate and efficient secondary analysis of large-scale immunological data.^10^

### Construction of the prognostic model

Differential gene analysis on all transcriptome profiling data of PDAC and normal pancreas samples from TCGA and GTEx databases were performed via the R software limma package (http://www.bioconductor.org/packages/release/bioc/html/limma.html), setting *P* value < 0.05 and |log_2_ fold-change|>1 as the cutoff values, and we used the pheatmap package in R to generate the heatmap. The differentially expressed IRGs were then extracted from the intersection of all differentially expressed genes and immune genes. By analyzing the clinical follow-up data from TCGA, the overall survival (OS) was selected as the primary endpoint. Univariable Cox regression analysis was used to select survival-associated genes (*P* < 0.05). Hazard ratio (HR) values were calculated by the coxph function of the stat package in R. Genes with HR values >1 were defined as high-risk genes. Genes with HR values <1 were defined as low-risk genes. Subsequently, we performed least absolute shrinkage and selection operator (LASSO) penalty Cox regression to avoid overfitting to develop the optimal signature. According to the expression data of these survival-associated genes and their coefficients, a prognostic prediction model was established. The formula was as follows: risk scores = ∑(Expi×βi) = expression data × coefficients.

### Evaluating the prognostic model

The risk score of PDAC patients in the training dataset (TCGA-PDAC) and the external validation dataset (ICGC-PACA-AU, ICGC-PACA-CA, GSE57495, and GSE62452) were estimated using the above formula. Meta-ICGC cohort was combined from two ICGC cohorts. Meta-GSE cohort was combined from two GEO cohorts. Meta-ICGC + GSE cohort was combined from two ICGC cohorts and two GEO cohorts. The patients were then divided into high-risk and low-risk groups based on the median risk score value. Survival differences of these two groups were then examined. The prognostic value of the five-genes signature was evaluated by ROC curves. We performed univariate Cox regression analysis and multivariate Cox regression analysis to test whether the signature was independent of clinical factors, including age, gender, grade, and tumor node metastasis classification (TNM) stage.

### Estimation of tumor-infiltrating immune cells

Cell type identification by estimating the relative subsets of RNA transcripts (CIBERSORT) is a deconvolution algorithm and was used to estimate the relative abundance of 22 immune cell types in PDAC tissues.^11^ CIBERSORT was run with default parameters.

The estimate score, stromal score, and immune score were assessed by estimation of stromal and immune cells in malignant tumors using expression data (ESTIMATE) based on the gene expression signature using the “estimate” package.^12^

### Prediction of immunotherapy responsiveness

The tumor immune dysfunction and exclusion (TIDE) algorithm^13^ is a method of evaluating the immunotherapeutic response based on genome-wide expression profiles of pretreatment patients. The TIDE score and prediction of immunotherapy responsiveness of PDAC patients were calculated by the TIDE algorithm.

### Statistical analysis

The Wilcoxon test was used to examine the statistical differences of numerable variables, while the chi-square test was used to compare the category variables. A Kaplan–Meier curve was drawn by the “survival” package and “survminer” package in R. Moreover, univariate and multivariate Cox regression analyses were performed to test whether the signature was independent of clinical factors. The sensitivity and specificity for survival prediction of the gene signature were examined by ROC analysis. Statistical significance was considered as *P*-value < 0.05. All statistical analyses were calculated by the R programming language.

## Results

### Construction and evaluation of a prognostic signature through comprehensive immunogenomic analysis

**Figure 1.**
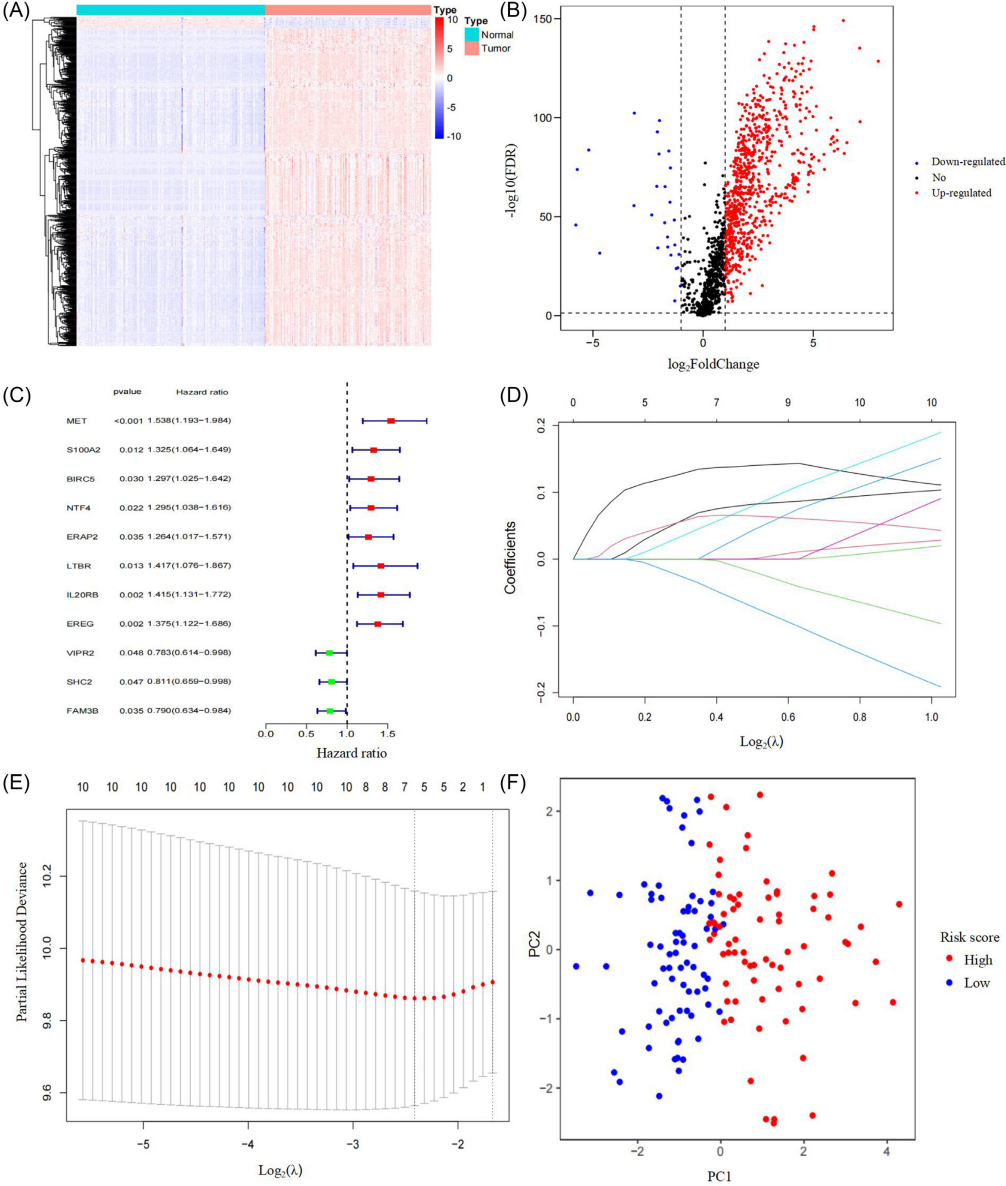
Construction of a prognostic signature through comprehensive immunogenomic analysis. (**A**) Heatmap showing differentially expressed immune-related genes. (**B**) Volcano plot showing differentially expressed immune-related genes. (**C**) Forest plot of the hazard ratios showing survival-associated IRGs. (**D**) LASSO coefficient profiles of the 11 immune genes in TCGA-PDAC. (**E**) A coefficient profile plot was produced against the log_2_(λ) sequence. (**F**) The expression patterns of different risk groups were analyzed by PCA using the five genes included in this model.

Firstly, 799 differentially expressed IRGs were screened between tumor and normal samples. Among them, 772 genes were up-regulated and 27 were down-regulated in tumor (Fig. 1A, B). Then through using univariate Cox regression analysis, we found 11 out of the 799 IRGs were significantly associated with OS (Fig. 1C). Subsequently, the most contributive variables were chosen based on the “lambda. min” standard by using LASSO penalty Cox analysis (Fig. 1D, E). A prognostic prediction model involving five genes (*MET, ERAP2, IL20RB, EREG*, and *SHC2*) was established by integrating the expression data and the coefficients of these prognostic genes. The formula is as follows: [expression level of *MET* × (0.13464)] + [expression level of *ERAP2* × (0.04507)] + [expression level of *IL20RB* × (0.06951)] + [expression level of *EREG* × (0.06361)] + [expression level of *SHC2* × (-0.03539)].

**Figure 2.**
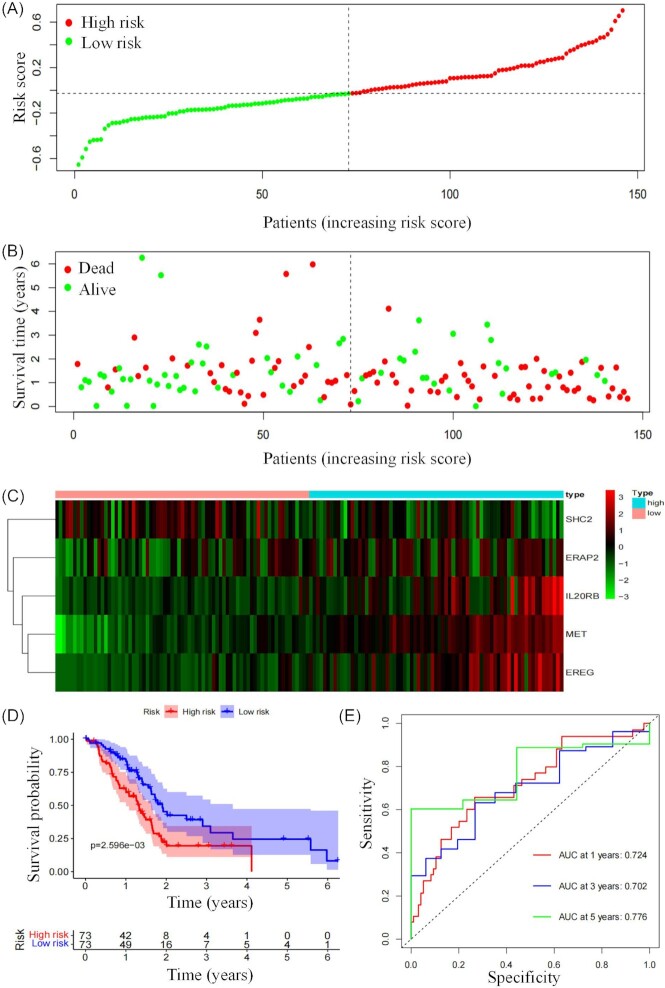
Evaluation of the prognostic signature. (**A**) Risk score distribution in PDAC patients. (**B**) Survival time of PDAC patients in ascending order of the risk score. (**C**) A heatmap of expression profiles of the five mRNAs. (**D**) Kaplan–Meier curves of OS stratified by the risk score in the low- and high-risk patients. (**E**) ROC curves of OS for the risk signature score at 1, 3, and 5 years.

Based on the median risk score, the PDAC patients were then divided into high-risk and low-risk groups. According to the expression levels of these 5 IRGs, principal component analysis (PCA) was further performed for each PDAC patient. The high-risk and low-risk groups obviously exhibited distinct expression patterns (Fig. 1F). Compared with the high-risk group, lower mortality rates were observed in the low-risk group (Fig. 2A and B). Additionally, the expression profiles of 5 mRNAs were displayed in the heatmap (Fig. 2C). Among these 5 prognostic-related genes, *SHC2* was positively correlated with OS, while *MET, ERAP2,IL20RB*, and *EREG* were negatively correlated with OS. It was also found that the prognostic signature can significantly distinguish the OS between the high-risk and low-risk groups (Fig. 2D). The area under curve (AUC) values of the ROC curve at 1, 3, and 5 years of survival were 0.724, 0.702, and 0.776, respectively, showing the model could effectively predict the clinical outcome of PDAC patients (Fig. 2E).

### Validation of the five-genes signature and comparison with other published signatures

**Figure 3.**
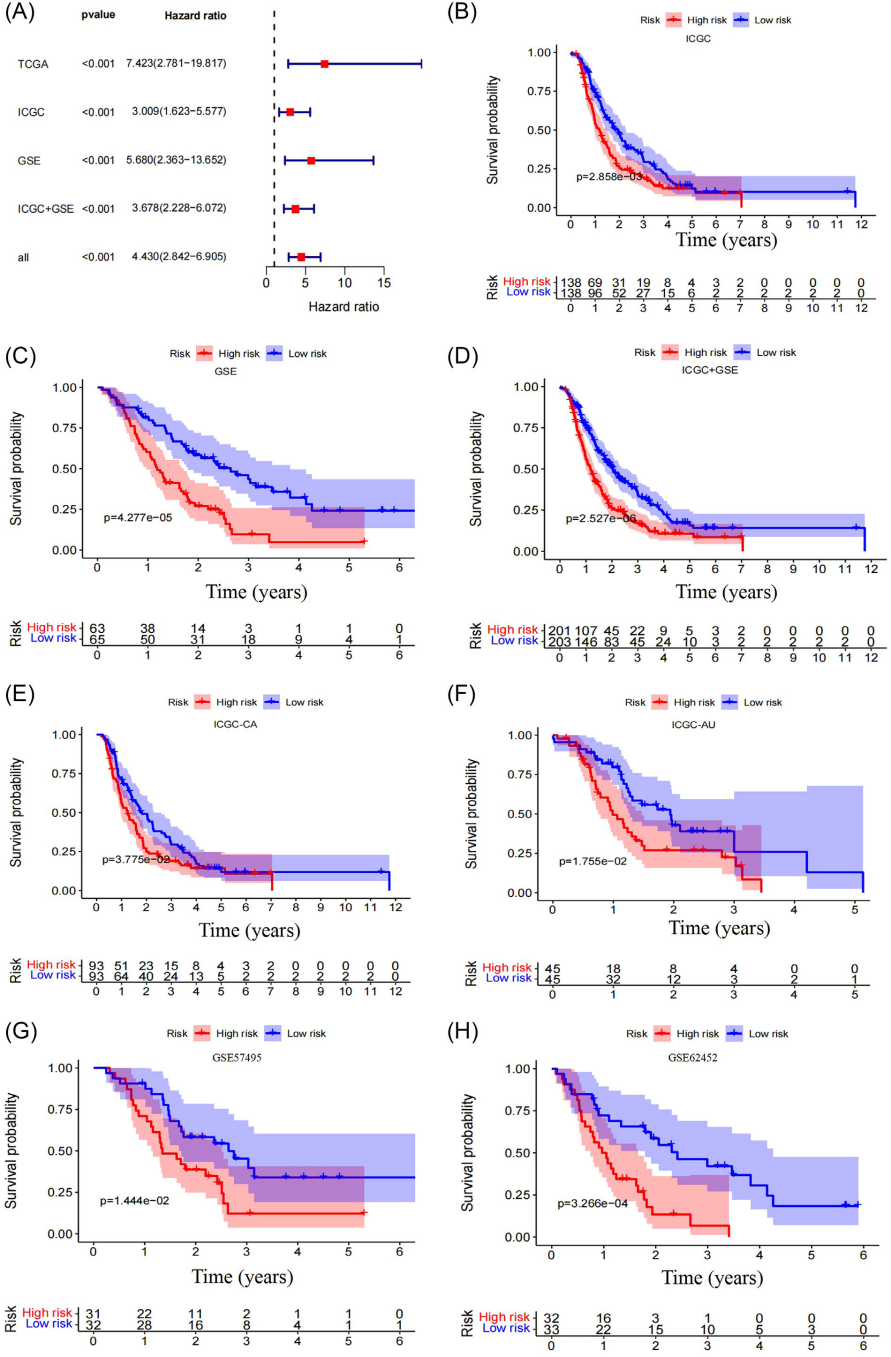
Validation of the five-genes signature. (**A**) A meta-analysis was performed using the prognostic results of the five-genes signature in different databases. (**B**–**D**) Kaplan–Meier curves were created to estimate OS for high- and low-risk groups from different databases. (**E**–**H**) Kaplan–Meier curves were created to estimate the OS for high- and low-risk groups from four independent cohorts.

**Figure 4.**
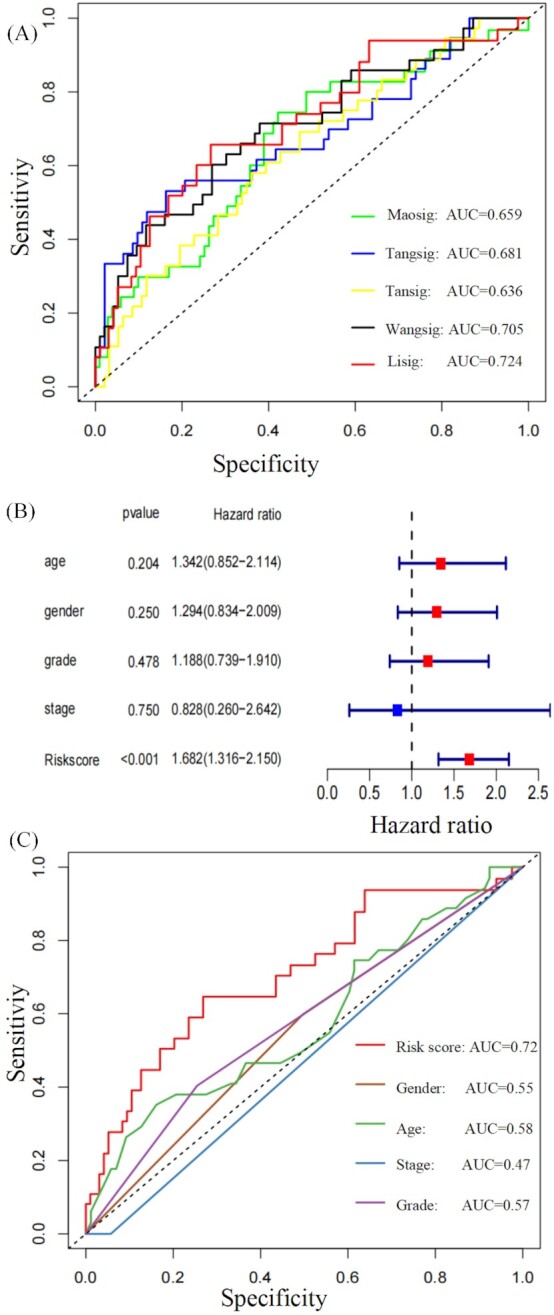
Comparison of the five-genes signature with other published signatures and traditional clinical factors. (**A**) Five-genes signature (Lisig) compared with four signatures (Maosig, Tangsig, Tansig, and Wangsig) published recently. (**B**) Multivariable analysis for risk score and clinical data. (**C**) Comparison of sensitivity and specificity of the ROC with traditional clinical factors.

In order to verify that the five-genes signature had a robust ability to distinguish the prognosis of PDAC patients, it was validated in two external databases (ICGC and GEO) consisting of four different cohorts ICGC-CA, ICGC-AU, GSE57495, and GSE62452. The five-genes signature performed excellently in all of the different databases, with HRs >1 (Fig. 3A). All Kaplan–Meier curves showed that the signature can significantly distinguish the OS between the high-risk and low-risk groups in TCGA, ICGC, and GEO databases (Fig. 3B–D). Additionally, the signature could also significantly distinguish the OS in different external cohorts (Fig. 3E–H). We further compared the prediction performance of the five-genes signature (Lisig) with four recently published immune-related gene signatures (Maosig, Tangsig, Tansig, and Wangsig) in PDAC and pancreatic cancer.^8,9,14,15^ As shown in Fig. 4A, the AUC at 1 year of OS for our signature is 0.724, which is higher than that of Maosig (0.659), Tangsig (0.681), Tansig (0.636), and Wangsig (0.705). These results suggest that our signature has better prognostic performance in predicting survival than that of four signatures recently published.

### Survival prediction of the five-genes signature was superior to traditional clinical indexes

To verify whether the signature was independent of sex, age, grade, and TNM stage covariables we performed multivariate Cox regression analyses. The result showed that risk scores (HR: 1.682, 95% confidence interval: 1.316−2.150, *P* < 0.001) had independent prognostic value (Fig. 4B). ROC curves further showed that risk score has higher sensitivity and specificity and is superior to traditional clinical indexes, including sex, age, grade, and TNM stage covariables (Fig. 4C).

### Relationship between the five-genes signature and immune infiltrating characteristics

**Figure 5.**
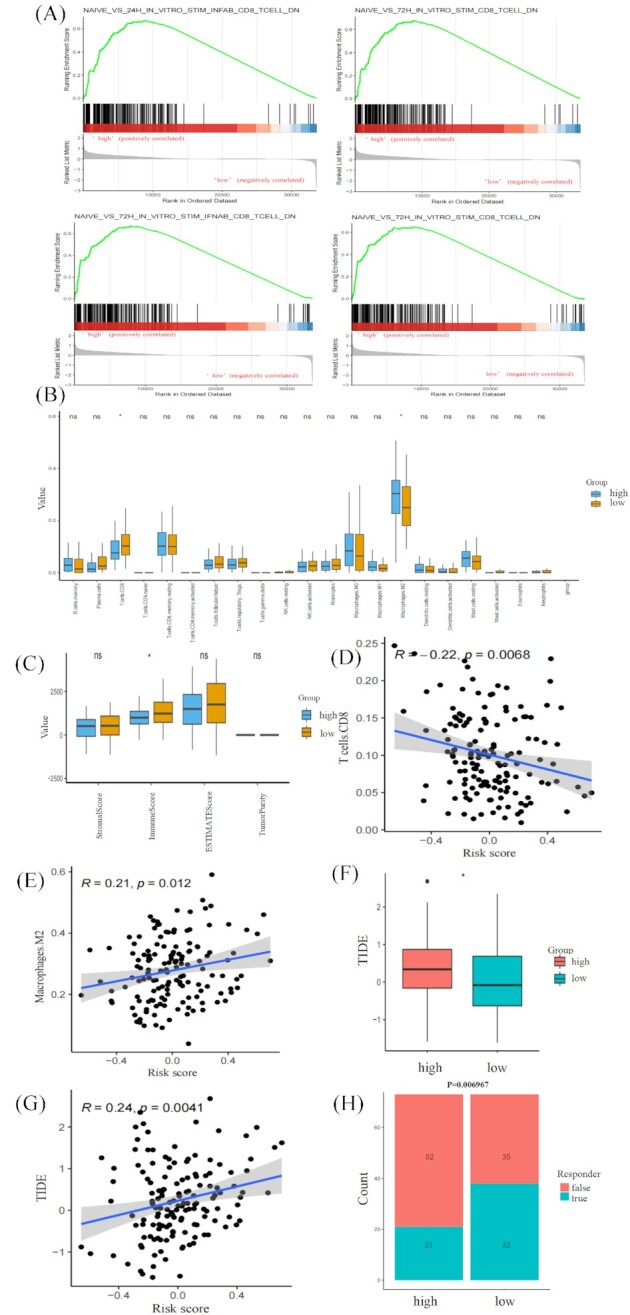
Relationship between the five-genes signature and immune infiltrating characteristics and immunotherapy response. (**A**) Significantly enriched pathways in the high-risk group of TCGA-PDAC. (**B**) Comparison of the 22 types of immune cells in the low- and high-risk groups estimated by the CIBERSORT algorithm. (**C**) Comparison of the estimate score, stromal score, and immune score in the low- and high-risk groups estimated by the ESIMATE algorithm. (**D**) Correlation analysis of risk score and abundance of CD8+ T cells by Pearson's correlation test. (**E**) Correlation analysis of risk score and abundance of M2-like macrophages by Pearson's correlation test. (**F**) Comparison of the TIDE scores. (**G**) Correlation analysis of risk score and TIDE scores by Pearson's correlation test. (**H**) Comparison of the proportion of predicted immunotherapeutic responders.

It is well known that the expression of immune-related genes in the tumor region could reflect the quality and abundance of immune infiltration in the TME. Thus, we further explored the relationship between the tumor immune infiltrating characteristics and five-genes signature. Gene Set Enrichment Analysis (GSEA) results showed that immunologic signature gene sets “NAIVE_VS_24H_IN_VITRO_STIM_INFAB_CD8_TCELL_DN”, “NAIVE_VS_24H_IN_VITRO_STIM_CD8_TCELL_DN”, “NAIVE_VS_72H_IN_VITRO_STIM_IFNAB_CD8_TCELL_DN”, and “NAIVE_VS_72H_IN_VITRO_STIM_CD8_TCELL_DN”, which reflect dysfunction of CD8 T cells, were enriched in the high-risk group (Fig. 5A). After quantifying 22 types of immune cells by CIBERSORT, we observed that the immune infiltration abundance of CD8+ T cells was significantly higher in the low-risk group compared with the high-risk group. M2-like macrophages in the high-risk group significantly increased compared with the low-risk group (Fig. 5B). The ESTIMATE algorithm further confirmed that the level of immune cells was significantly higher in the low-risk group (Fig. 5C). Correlation analysis showed that the risk scores were negatively correlated with the abundance of CD8+ T cells (Fig. 5D). Risk scores were highly positively correlated with the abundance of M2-like macrophages (Fig. 5E).

### Association of the five-genes signature with immunotherapy response

The TIDE algorithm assessed the predictive capability of the five-genes signature in the immunotherapeutic response. PDAC patients in the high-risk group have significantly higher TIDE scores, indicating more immune dysfunction in the high-risk group (Fig. 5F). Additionally, risk scores were significantly positively correlated with TIDE scores (Fig. 5G), confirming a close association between the five-genes signature and immunotherapy response. The patients in the low-risk group have a relatively higher proportion of immunotherapeutic responders compared with those in the high-risk group (Fig. 5H). Thus, we speculate that lower immunotherapy response in the high-risk group may be due to dysfunction and low abundance of immune infiltration in the TME.

## Discussion

PDAC is one of the most lethal neoplasms. The 5-year OS rate is <5% and the median survival duration is <6 months.^16^ Given the importance of immune genes in the progression of cancer, it is crucial to identify immune-related biomarkers for assessing the prognosis of PDAC patients.^17,18^ For predicting the OS of PDAC, integrating multigene signals through reliable algorithms will be better than single molecules. In our study, we have constructed a prognostic model to assess the clinical outcome of PDAC and comprehensively validated it in two external PDAC databases (ICGC and GEO). Moreover, comparing the prediction performance with four recently published immune-related genes signatures, we further demonstrated that our signature has better prognostic performance in predicting survival. ROC curves further showed that risk score has higher sensitivity and specificity and is superior to traditional clinical factors, including sex, age, grade, and TNM stage covariables. Additionally, the risk score had independent predictive value for OS in PDAC patients: the AUC for 1-year OS was 0.724. These results indicate that this five-genes signature for predicting the prognosis of PDAC patients has great applicability and stability.

It is well known that expression of immune-related genes in the tumor region could reflect the quality and abundance of immune infiltration in the TME. The characteristics of immune infiltration affect antitumor efficiency. It is reported that the abundance of T cell infiltration in the TME varies remarkably in PDAC patients.^19^ CD8+ cytotoxic T cells or tumor-infiltrating lymphocytes are associated with favorable survival in PDAC.^20,21^ M2 phenotype of macrophages is a suppressor of anti-tumor immunity in the TME.^22^ PDAC patients with a higher abundance of M2-like macrophages experienced adverse OS outcomes.^23^ In this study, the PDAC patients in the high-risk group had a lower fraction of CD8+ T cells and higher M2-like macrophages than those in the low-risk group. Furthermore, analysis using the ESTIMATE algorithm showed that the low-risk group was correlated with higher immune scores. These results suggest that the low-risk group might be “hot tumor”, partially explaining the prognostic value of this signature.

Recently, immunotherapy has attracted wide attention in clinical treatment for cancer. It brings a new dawn for improving the prognosis of cancer patients.^24^ Considering the low-risk group as “hot tumor”, suggests that immunotherapy might be effective. The relationship between the five-genes signature and potential immunotherapy response was further investigated. Significantly higher TIDE scores were observed in the high-risk group, indicating more tumor immune dysfunction. Additionally, the PDAC patients in the low-risk group have a relatively higher proportion of immunotherapeutic responders. GSEA results also showed that immunologic signature gene sets that reflect dysfunction of CD8+ T cells were highly enriched in the high-risk group. In addition, patients in the high-risk group had lower CD8+ T cells and higher M2-like macrophages. We speculate that the lower immunotherapy response in the high-risk group might be due to low abundance and more dysfunction of immune infiltration in the TME. Therefore, we propose that immunotherapy could be attempted for advanced PDAC patients in the low-risk group after gemcitabine treatment failure.

Nevertheless, our study has some limitations. Firstly, we lacked validation in our clinical cohorts, despite having constructed and evaluated the prognostic signature in two external databases. Moreover, the downstream molecular mechanisms of these five genes need to be further explored. Further *in vivo* and *in vitro* studies are urgently needed to improve current therapeutic practice in PDAC.

## Conclusions

Collectively, we constructed a novel prognostic model that has independent prognostic value and comprehensively validated it in two external PDAC databases (ICGC and GEO). Our signature has better prognostic performance in predicting survival than those recently published. This five-genes signature could predict immune infiltration characteristics: abundance and dysfunction of CD8+ T and M2-like macrophages. Our model found that PDAC patients in the low-risk group might be more responsive to immunotherapy.

## Data Availability

The data used to support the findings of this study are available from the corresponding author on reasonable request.
